# Detection of pathogens by real-time PCR in adult patients with acute exacerbation of bronchial asthma

**DOI:** 10.1186/s12890-017-0494-3

**Published:** 2017-11-22

**Authors:** Yutaka Yoshii, Kenichiro Shimizu, Miyuki Morozumi, Naoko Chiba, Kimiko Ubukata, Hironori Uruga, Shigeo Hanada, Hiroshi Wakui, Shunsuke Minagawa, Hiromichi Hara, Takanori Numata, Keisuke Saito, Jun Araya, Katsutoshi Nakayama, Kazuma Kishi, Kazuyoshi Kuwano

**Affiliations:** 1 Division of Respiratory Diseases, Department of Internal Medicine, The Jikei University School of Medicine, 3-25-8 Nishi-Shimbashi, Minato-ku, Tokyo, 105-8461 Japan; 2 Department of Infectious Diseases, Keio University School of Medicine, 35 Shinanomachi, Shinjuku-ku, Tokyo, 160-8582 Japan; 3 Department of Respiratory Medicine, Respiratory Center, Toranomon Hospital, 2-2-2 Toranomon, Minato-ku, Tokyo, 105-8470 Japan; 4 Division of Respiratory Diseases, Department of Internal Medicine, The Jikei University Daisan Hospital, 4-11-1 Izumihoncho, Komae-shi, Tokyo, 201-8601 Japan

**Keywords:** Acute exacerbation, Bronchial asthma, Pathogen, Real-time polymerase chain reaction, Risk factor

## Abstract

**Background:**

Respiratory tract infection is a major cause of acute exacerbation of bronchial asthma (AEBA). Although recent findings suggest that common bacteria are causally associated with AEBA, a comprehensive epidemiologic analysis of infectious pathogens including common/atypical bacteria and viruses in AEBA has not been performed. Accordingly, we attempted to detect pathogens during AEBA by using real-time polymerase chain reaction (PCR) in comparison to conventional methods.

**Methods:**

We prospectively enroled adult patients with AEBA from August 2012 to March 2014. Infectious pathogens collected in nasopharyngeal swab and sputum samples were examined in each patient by conventional methods and real-time PCR, which can detect 6 bacterial and 11 viral pathogens. The causal association of these pathogens with AEBA severity and their frequency of monthly distribution were also examined.

**Results:**

Among the 64 enroled patients, infectious pathogens were detected in 49 patients (76.6%) using real-time PCR and in 14 patients (21.9%) using conventional methods (*p* < 0.001). Real-time PCR detected bacteria in 29 patients (45.3%) and respiratory viruses in 28 patients (43.8%). *Haemophilus influenzae* was the most frequently detected microorganism (26.6%), followed by rhinovirus (15.6%). Influenza virus was the significant pathogen associated with severe AEBA. Moreover, AEBA occurred most frequently during November to January.

**Conclusions:**

Real-time PCR was more useful than conventional methods to detect infectious pathogens in patients with AEBA. Accurate detection of pathogens with real-time PCR may enable the selection of appropriate anti-bacterial/viral agents as a part of the treatment for AEBA.

## Background

Bronchial asthma is a common chronic inflammatory disease of the airways characterised by variable and recurring symptoms, reversible airflow obstruction, and bronchospasm [1]. Acute exacerbation of bronchial asthma (AEBA) is the acute worsening of clinical symptoms caused by various factors, including respiratory infections, which are associated not only with the deterioration of lung function but also with hospitalisation or death [2–4]. Although the overall in-hospital mortality of American asthmatic patients over a recent 10-year period was 0.97%, it reached 9.8% in patients requiring mechanical ventilation [5]. Moreover, the in-hospital mortality in Japan was 1.2% [6]. Thus, because bronchial asthma is likely still a fatal disease especially in the setting of AEBA, detecting the etiologic agents for AEBA can be of considerable clinical importance.

Respiratory tract infection is one of the major causes of AEBA, and thus respiratory viruses and atypical bacteria have attracted attention as causative pathogens of AEBA [7–14]. In particular, rhinovirus (RV) and *Mycoplasma pneumoniae* are reported to be of critical importance in AEBA [8, 11, 15–17]. However, common bacteria have received much less attention than viruses or atypical bacteria in AEBA because their pathogenic role and frequency during AEBA are largely obscure. However, several recent reports have shown that infections with bacteria such as *Haemophilus influenzae* and *Streptococcus pneumoniae* may have a pathogenic role in the development of AEBA [18, 19]. Meanwhile, comprehensive epidemiologic analysis of infectious pathogens, including these common bacteria, atypical bacteria, and viruses, during AEBA has not been performed. Moreover, a causal link between these pathogens and disease severity and the seasonality of AEBA remains elusive.

Polymerase chain reaction (PCR) technology has attracted attention over the last 20 years because it can simultaneously detect not only common bacteria but also viruses and atypical bacteria, which are hardly detectable by conventional methods. We have already reported the advantage of using real-time PCR method to detect various pathogens in patients with adult community-acquired pneumonia and exacerbation of chronic obstructive pulmonary disease [20, 21]. Thus, we thought to apply comprehensive real-time PCR analysis to more accurately detect the infectious pathogens associated with AEBA.

In the present study, we have attempted to detect the pathogens associated with AEBA by using comprehensive real-time PCR of nasopharyngeal swab (NPS) and sputum samples from adult patients with AEBA in comparison to conventional methods such as sputum culture. The causal association of these pathogens with AEBA severity and their frequency of monthly distribution were also examined.

## Methods

### Study population and definitions

From August 2012 to March 2014, the present study prospectively recruited Japanese patients with AEBA aged 20 years or more at the outpatient clinic and emergency room of The Jikei University Hospital, The Jikei University Daisan Hospital, and Toranomon Hospital. Only patients diagnosed by respiratory medicine specialists as having AEBA were included. AEBA was defined as any sustained worsening beyond the patients’ baseline condition within 7 days of onset of any respiratory symptom that required a change in regular medications and/or hospital treatment. The severity of AEBA was defined based on The Global Initiative for Asthma guidelines [1]. The patients in whom environmental factors and stress were strongly suspected as the causes of AEBA were excluded, as were those with the complication of obvious pneumonia. After written informed consent was obtained from each patient, NPS and sputum were collected by a physician or nurse.

### Data collection

Data regarding patient age, sex, asthma onset, smoking history, influenza vaccination within 6 months of recruitment, underlying disease, serum total immunoglobulin E (IgE), pulmonary function testing during a stable bronchial asthma state, and compliance to inhaled corticosteroid (ICS) were obtained from the patients’ medical records. We also recorded the requirement for hospitalisation and the frequency, severity of attack, and monthly distribution of AEBA.

### Multiplex real-time PCR

Multiplex real-time PCR for clinical samples was performed as described previously [20, 21]. Briefly, sputum samples were homogenised with an enzymatic reagent (Sputazyme; KYOKUTO, Tokyo, Japan). NPS was suspended in 0.5 mL of PPLO broth (Difco, Detroit, MI, USA) for the detection of bacteria including *M. pneumoniae* (see next section). After extracting DNA and RNA from these samples, real-time PCR was performed with the use of two commercial PCR kits that were developed on the basis of previous papers co-authored by some of the present authors and as described therein [22, 23]. These kits can detect the viruses and bacteria mentioned below, which are representative causative pathogens of respiratory infections in Japan [20–24].

The first is a Cycleave PCR kit (catalogue no. CY216; Takara Bio, Shiga, Japan) that can detect 11 respiratory viruses: RV; influenza virus types A and B; respiratory syncytial virus subgroup A (RSV A) and subgroup B (RSV B); parainfluenza virus type 1 (PIV1), type 2 (PIV2), and type 3 (PIV3); human metapneumovirus; adenovirus; and human bocavirus. The sensitivity of PCR for each virus was approximately 10 plaque-forming units per well.

The second is a Cycleave PCR kit (catalogue no. CY 214; Takara Bio) that can detect six bacterial pathogens: *S. pneumoniae*, *H. influenzae*, *M. pneumoniae*, *Chlamydophila pneumoniae*, *S. pyogenes*, and *Legionella pneumophila*. Results by the real-time PCR were recorded as positive if a pathogen was detected in at least one of two samples.

### Conventional methods of pathogen detection in AEBA

Conventional methods performed for pathogen detection included NPS and sputum cultures and serological tests. For bacterial cultures, the PPLO broth in which the NPS was suspended was incubated at 37 °C for the detection of *M. pneumoniae*. Aliquots (5 μL) of the PPLO broth suspension and the homogenised sputum sample were inoculated on sheep blood, chocolate, mannitol salt, and modified Drygalski agar plates (all from Nippon Becton Dickinson, Tokyo, Japan). Blood and chocolate agar plates were incubated at 37 °C for 20 h in an atmosphere containing 5% CO_2_. Colonies grown on each agar plate were picked up and subjected to detection of bacterial species by routine methods. Detection of *S. pneumoniae* and *L. pneumophila* antigens was done with urine assays (Binax, Inc., Portland, ME, USA), and a NPS sample was collected for a rapid antigen test of influenza virus (Tauns Laboratories, Inc., Numazu, Shizuoka, Japan) if thought necessary. Except for resident bacteria of the mouth, a bacterium or virus was defined as a causative pathogen if it was detected by at least 1 of the sampling methods.

### Statistical analysis

The chi-square test and Fisher’s exact test were used for categorical data as appropriate. McNemar’s test was used to compare groups with categorical variables. Univariate analyses were conducted, and then age, sex, and variables associated with a *p* value of <0.1 from these analyses were included in the multivariate analysis. Odds ratio (OR) and 95% confidence intervals (CIs) were synchronously calculated. A *p* value of <0.05 was considered statistically significant. We used the Microsoft Excel 2010 for Statistics (Microsoft, Redmond, WA, USA) and Stat View 5.0 (Abacus Concepts Inc., Berkley, CA, USA) software programs to perform the statistical analyses.

## Results

### Patient characteristics

This study included 64 patients (23 men, 41 women; mean age, 55.1 years) from August 2012 to March 2014 (Table 1). Twenty-five patients (39.1%) were current or previous smokers. Twenty patients (31.3%) had received influenza vaccination. ICS was used regularly in 45 patients (70.3%). Forced expiratory volume in the first second (FEV_1.0_) was less than 70% in 14 patients (35.0%). Overall, AEBA occurred at a mean frequency of 1.5 (range, 1–5) times per year; 18 patients (28.1%) had a severe attack, and 15 patients (23.4%) were admitted to the hospital. The underlying diseases are summarised in Table 1.

**Table 1:** Characteristics of the patients (*n* = 64)

	N (%)
Mean age, yr. ± SD (range)	55.1 ± 18.1 (21–92)
Male	23 (35.9)
Onset of asthma	
Paediatric	23 (35.9)
Adult	41 (64.1)
Smoking history	
Never smoker	39 (60.9)
Current or previous smoker	25 (39.1)
Influenza vaccination, yes	20 (31.5)
Underlying disease	
Sinusitis	8 (12.5)
Allergic rhinitis	3 (4.7)
COPD	6 (9.4)
GERD	5 (7.8)
DM	6 (9.4)
Mean serum total IgE (IU/ml) ± SD (range)^a^	463.9 ± 976 (0–4980)
FEV_1.0_ < 70%^b^	14 (35.0)
Regular use of ICS^c^	45 (90.0)
Median treatment step, (range)	2 (1–4)
Frequency of exacerbations, number/yr. (range)	1.48 (1–5)
Severity of attack^d^	
Mild	28 (43.8)
Moderate	18 (28.1)
Severe	18 (28.1)
Hospitalisation	15 (23.4)

Data are presented as the number (percentage) of patients

COPD, chronic obstructive pulmonary disease; DM, diabetes mellitus; FEV_1.0_, forced expiratory volume1.0 (sec); GERD, gastroesophageal reflux disease; ICS, inhaled corticosteroids

^a^Total number of patients with a measured IgE value: 36

^b^Total number of patients with a pulmonary function test: 40

^c^Total number of patients regularly treated with ICS: 50

^d^Severity of attack was defined according to the 2012 GINA guideline [1]

### Pathogens detected using each method

The rate of pathogen detection by real-time PCR in all 64 patients was 76.6% (49 patients), whereas that of pathogen detection by the conventional methods was 21.9% (14 patients, *p* < 0.001; Table 2). *H. influenzae* was detected by real-time PCR analysis most frequently in 17 patients (26.6%), followed by RV in 10 (15.6%), influenza virus in 9 (14.1%), and *S. pneumoniae* in 6 (9.4%). All *H. influenzae* and *S. pneumoniae* detected by conventional methods were also detected by real-time PCR. Real-time PCR showed that 39 pathogens (61.0%) were considered to cause a single-microbe infection, whereas 10 pathogens (15.6%) caused polymicrobial infections.

**Table 2:** Pathogens identified in patients with AEBA

Pathogen	Total	Real-time PCR			Conventional methods	*p*-Value^a^
		NPS	Sputum	Total		
Any pathogen detected, n (%)	50 (78.1)	32 (50.0)	41 (64.1)	49 (76.6)	14 (21.9)	<0.001
Single pathogen	39 (60.9)	28 (43.8)	36 (56.3)	39 (60.9)	13 (20.3)	<0.001
Mixed pathogens	11 (17.2)	4 (6.3)	5 (7.8)	10 (15.6)	1 (1.6)	0.008
Viral pathogens, n (%)	28 (43.8)	25 (39.1)	17 (26.6)	28 (43.8)	7 (10.9)	<0.001
Influenza virus	9 (14.1)	8 (12.5)	4 (6.3)	9 (14.1)	7 (10.9)	0.480
Influenza virus A	6 (9.4)	6 (9.4)	2 (3.1)	6 (9.4)	5 (7.8)	1.000
Influenza virus B	3 (4.7)	2 (3.1)	2 (3.1)	3 (4.7)	2 (3.1)	1.000
Rhinovirus	10 (15.6)	9 (14.1)	7 (10.9)	10 (15.6)	NA	–
Respiratory syncytial virus	4 (6.3)	3 (4.7)	3 (4.7)	4 (6.3)	NA	–
Subgroup A	3 (4.7)	2 (3.1)	2 (3.1)	3 (4.7)	NA	–
Subgroup B	1 (1.6)	1 (1.6)	1 (1.6)	1 (1.6)	NA	–
Parainfluenzae virus	5 (7.8)	5 (7.8)	3 (4.7)	5 (7.8)	NA	–
Subtype 1	3 (4.7)	3 (4.7)	1 (1.6)	3 (4.7)	NA	–
Subtype 3	2 (3.1)	2 (3.1)	2 (3.1)	2 (3.1)	NA	–
Bacterial pathogens, n (%)	30 (46.9)	10 (15.6)	27 (42.2)	29 (45.3)	8 (12.5)	<0.001
*Haemophilus influenzae*	17 (26.6)	4 (6.3)	17 (26.6)	17 (26.6)	2 (3.1)	<0.001
*Streptococcus pneumoniae*	6 (9.4)	3 (4.7)	6 (9.4)	6 (9.4)	3 (4.7)	0.248
*Streptococcus pyogenes*	2 (3.1)	2 (3.1)	2 (3.1)	2 (3.1)	1 (1.6)	1.000
*Mycoplasma pneumoniae*	5 (7.8)	1 (1.6)	4 (6.3)	5 (7.8)	0	0.074
*Chlamydophila pneumoniae*	1 (1.6)	1 (1.6)	0	1 (1.6)	NA	–
*Moraxella catarrhalis*	2 (3.1)	ND	ND	ND	2 (3.1)	–
No pathogen detected	14 (21.9)	32 (50.0)	23 (35.9)	15 (23.4)	50 (78.1)	–

Data are presented as the number (percentage) of patients

*AEBA* acute exacerbation of bronchial asthma, *NA* not applicable, *NPS* nasopharyngeal swab, *PCR* polymerase chain reaction

^a^McNemar’s test was used to compare groups for total pathogens found by PCR and conventional methods

^b^Some overlap exists

Conventional methods detected 2 patients with *Moraxella catarrhalis*. However, all microorganisms, except for *M. catarrhalis*, that were detected using conventional methods were also detected using real-time PCR.

*M. pneumoniae* was detected in 5 patients by PCR, but it was not detected in these patients by the culture method using PPLO broth. However, *L. pneumophila* was not detected in any of the patients by either method.

### Detection of pathogens using both methods

**Fig. 1:**
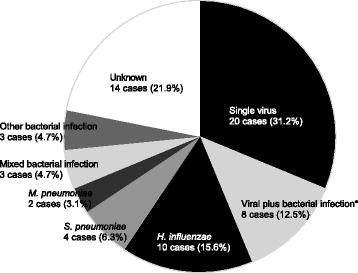
Percentages of pathogens detected by comprehensive real-time polymerase chain reaction and conventional methods. *Influenza virus + *H. influenzae*, 3 cases (4.7%); rhinovirus + *H. influenzae*, 2 cases (3.1%); respiratory syncytial virus + *H. influenzae*, 1 case (1.6%); influenza virus + *M. pneumoniae*, 1 case (1.6%); influenza virus + *H. influenzae* + *S. pneumoniae*, 1 case (1.6%)

The respective percentages of detected pathogens by comprehensive real-time PCR and conventional methods are shown in Fig. 1. By the use of both methods, 62 pathogens were detected in 50 patients (78.1%). The infectious etiology was viral only in 20 patients (31.3%) and both viral and bacterial in 8 patients (12.5%). In addition, the most common single bacterial pathogen was *H. influenzae*, which was detected in 10 patients (15.6%), followed by *S. pneumoniae* in 4 patients (6.3%), and *M. pneumoniae* in 2 patients (3.1%).

### Relationship between the development of influenza infection and influenza vaccination

Among the 44 patients who had been not vaccinated against the influenza virus, 9 patients (20.5%) developed influenza infection, whereas none of the 20 patients (0%) who received the vaccination developed influenza infection (*p* = 0.047 by Fisher’s exact test).

### Risk factors associated with severe AEBA

The results of univariate and multivariate analyses of AEBA risk factors are shown in Tables 3 and 4, respectively. Infection caused by influenza virus was significantly associated with severe exacerbation (OR 7.107; 95% CI 1.511–33.43; *p* = 0.013). Other respiratory viruses, *H. influenzae*, and *S. pneumoniae* were not significant variables affecting the severity of AEBA.

**Table 3:** Univariate analysis of risk factors for severe AEBA

Variable	Severity of asthma attack		*p-*Value
	Mild/Moderate (*n* = 46)	Severe (*n* = 18)	
Age > 50 years	22 (47.8)	14 (77.8)	0.125
Male sex	15 (32.6)	8 (44.4)	0.375
Smoking history	19 (41.3)	6 (33.3)	0.557
Regular use of ICS	35 (76.1)	10 (55.6)	0.106
Bacterial agent	22 (47.8)	8 (44.4)	0.807
*H. influenzae*	13 (28.3)	4 (22.2)	0.758
*S. pneumoniae*	3 (6.5)	3 (16.7)	0.807
*M. pneumoniae*	4 (8.7)	1 (5.6)	> 0.999
Viral agent	17 (37.0)	1 (5.6)	0.080
Influenza virus	3 (6.5)	6 (9.4)	0.012
Rhinovirus	8 (17.4)	2 (11.1)	0.712

Data are presented as the number (percentage) of patients. *AEBA* acute exacerbation of bronchial asthma, *ICS* inhaled corticosteroids

**Table 4:** Multivariate analysis of risk factors for severe AEBA

Variable	Logistic multivariate analysis	
	Odds ratio (95% CI)	*p*-Value
Age > 50 years	1.664 (0.464–5.964)	0.434
Male sex	1.500 (0.452–4.976)	0.508
Influenza virus	7.107 (1.511–33.43)	0.013

*AEBA* acute exacerbation of bronchial asthma, *CI* confidence interval

### Monthly distribution and pathogens in AEBA

**Fig. 2:**
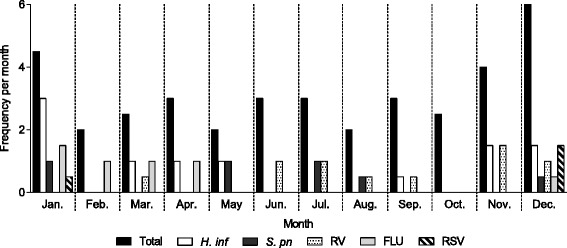
Monthly distribution and frequent pathogens in acute exacerbations of bronchial asthma. FLU, influenza virus; *H. inf*, *Haemophilus influenza*; RV, rhinovirus; RSV, respiratory syncytial virus; *S. pn*, *Streptococcus pneumoniae*

The monthly distributions of frequencies and characteristic pathogens of AEBA are shown in Fig. 2. AEBA clearly followed a seasonal pattern, with the highest monthly rates occurring from November to January, during which 14.5 instances (38.7%) of exacerbation were observed.

## Discussion

The present study of adult patients with AEBA showed that in comparison to conventional methods, real-time PCR using NPS and sputum samples could efficiently detect different types of microorganisms except for *M. catarrhalis*, for which primers were not included in the PCR kit. The detection rate of real-time PCR was significantly higher than that of the conventional methods (76.6% vs. 21.9%, *p* < 0.001). Moreover, real-time PCR could efficiently detect mixed pathogens at a 10-fold higher rate than the conventional methods (15.6% vs. 1.6%, *p* = 0.008).

Consistent with previous studies in which the detection rate ranged from 41 to 55% [8, 10], the detection rate of respiratory viruses by real-time PCR was 43.8% in the present study. Intriguingly, viruses were more frequently detected from NPS samples than from sputum samples (39.1% vs. 26.6%), suggesting the nasopharyngeal-dominant distribution of respiratory virus in the setting of AEBA. However, common bacteria were more frequently detected by real-time PCR using sputum samples rather than NPS samples or conventional methods (45.3% vs. 17.2% vs. 12.5%). Real-time PCR using sputum samples may be suitable for the detection of bacteria associated with AEBA. Especially, in the situations in which antibiotics have already been administered for AEBA patients, real-time PCR using sputum samples may also be useful in the detection of associated bacteria because the pathogen detection rate by real-time PCR was higher than that by sputum culture in patients with community-acquired pneumonia receiving prior antibiotic treatment [20].

We extended the application of real-time PCR to include not only viruses and atypical bacteria but also common bacterial pathogens. As a result, *H. influenzae* was most frequently detected at 26.6%, followed by *S. pneumoniae* at 9.4%. For these two bacteria, real-time PCR could detect all bacteria also detected by the conventional methods. In contrast, a previous study showed that *H. influenzae* was observed in 30% of the stable asthmatic patients by real-time PCR using NPS samples [25]. However, it has also been reported that airway colonisation with potentially pathogenic bacteria such as *Haemophilus* or *Streptococcus* genera in stable asthmatics is associated with severe airway obstruction and neutrophilic airway inflammation (so called steroid-resistant neutrophilic asthma) [26, 27]. Hence, because we have speculated that common bacteria play a role in triggering the exacerbation of neutrophilic asthma, we considered that the common bacteria detected in patients with AEBA were causative pathogens. Indeed, we found that the common bacteria detected at the onset of AEBA were not detected by re-examination with real-time PCR after the improvement of AEBA in several cases in the present study (data not shown), although re-examination was not performed in all cases. This finding may support our speculation that these common bacteria trigger AEBA as the causative pathogens. Further studies of the influence of common bacteria on AEBA are needed from the points of view of infectious and allergic mechanisms.

Some studies have shown clear benefits from antibiotic therapy for AEBA triggered by bacteria, resulting in a reduction of asthmatic symptoms, improvement of lung function, and control of bronchial hyperreactivity [28, 29]. Moreover, antibiotic therapy may also be effective in neutrophilic asthma caused by common bacteria [26, 27]. However, the role of empiric antibiotic therapy without clear evidence of a bacterial trigger for severe AEBA is unclear [30]. Although there is an urgent need for further studies investigating the beneficial role of antibiotic treatment in AEBA triggered by bacteria, we believe that the accurate detection of bacteria using real-time PCR can lead to the appropriate use of antibiotics for AEBA patients with bacterial infection.

Influenza virus was the sole factor that was significantly associated with severe AEBA in the present study. In line with our findings, Wark et al. reported that influenza infection resulted in severe and refractory AEBA requiring intensive care [9]. Influenza virus causes cytopathic damage to airway epithelial cells, and these changes can impair the functional integrity of the epithelium and airway smooth muscle by inducing NF-κB–mediated cytokine release [31]. In addition, loss of functional integrity of the epithelial layer enhances mucosal permeability and may increase the exposure of inflammatory and antigen-presenting cells to other pathogens, allergens, and irritants. Consequently, influenza virus can cause severe AEBA.

The morbidity of influenza infection in the unvaccinated patients with AEBA was significantly higher than that in the vaccinated patients in the present study (20.5% vs. 0%, *p* = 0.047). The vaccination should be strongly considered in asthmatic patients to help prevent AEBA, although full elucidation of the degree of protection offered by vaccination against AEBA is awaited [32]. In asthmatic patients suspected of developing influenza infection despite the vaccination, it is likely that the accurate diagnosis of influenza virus by real-time PCR may result in the earlier initiation of anti-influenza virus therapy to prevent the development of severe AEBA.

Our study showed that the peak instances of AEBA, which occurred during November to January, were associated with the high frequent appearance of RV, influenza virus, RSV, and *H. influenzae*. Consistent with our results, previous research from western countries has already revealed that these viruses contribute to adult AEBA in the winter season [33–35]. A French article also reported that bronchopulmonary infectious diseases including asthma related to *H. influenzae* occurred mainly in the winter and spring [36]. To the best of our knowledge, the present study is the first to evaluate the seasonal pattern of these pathogens associated with AEBA in Japan. Moreover, similar to our results, Johnston reported that AEBA in Canadian adults is at its highest average annual level during the Christmas period [37]. The similar results from western countries and Japan emphasise the importance of paying special attention to the development of AEBA during the winter season.

The present study has some limitations. First, a relatively small number of samples were evaluated. Nevertheless, this was a prospective, multi-centre study. Second, re-examination with real-time PCR was performed in only some patients in the convalescent stage after AEBA and not in all patients. To accurately distinguish true active infection by the detected pathogens from colonisation and to more clearly understand the role of the causative pathogens in AEBA, further study using samples from both the acute and convalescent stages is required. Third, *M. catarrhalis* was not detected by the comprehensive real-time PCR because primers for this bacterium were not included in this real-time PCR. Hence, when no pathogens are detected by real-time PCR in patients with AEBA, other pathogens including *M. catarrhalis* should be suspected, although the frequency of their detection may be low. Fourth, because the study period was relatively short, the results were influenced by annual variations in the incidence of specific infections.

## Conclusions

Comprehensive real-time PCR was more useful than conventional methods for the detection of pathogenic microorganisms that aggravate AEBA. *H. influenzae* was the most frequent pathogen, whereas influenza virus was the significant pathogen associated with severe AEBA. Accurate detection of pathogenic microorganisms by real-time PCR is highly recommended to ensure the appropriate use of antibiotic and antiviral therapies.

## Abbreviations

AEBA: Acute exacerbation of bronchial asthma
CI: Confidence interval
FEV_1.0_: Forced expiratory volume in the first second
ICS: Inhaled corticosteroid
IgE: Immunoglobulin E
NPS: Nasopharyngeal swab
OR: Odds ratio
PCR: Polymerase chain reaction
PIV: Parainfluenza virus
RSV: Respiratory syncytial virus
RV: Rhinovirus
